# Strong Static Magnetic Fields Increase the Gel Signal in Partially Hydrated DPPC/DMPC Membranes

**DOI:** 10.3390/membranes5040532

**Published:** 2015-09-29

**Authors:** Jennifer Tang, Richard J. Alsop, Karin Schmalzl, Richard M. Epand, Maikel C. Rheinstädter

**Affiliations:** 1 Department of Physics and Astronomy, McMaster University, Hamilton, ON, L8S 4M1, Canada; E-Mails: tangj43@mcmaster.ca (J.T.); alsoprj@mcmaster.ca (R.J.A.); 2 JCNS, Forschungszentrum Jülich, Outstation at ILL, 38042 Grenoble, France; E-Mail: schmalzl@ill.eu; 3 Department of Biochemistry and Biomedical Sciences, McMaster University, Hamilton, ON, L8S 4K1, Canada; E-Mail: epand@mcmaster.ca

**Keywords:** lipid membranes, effect of magnetic fields on membranes, neutron diffraction, membrane electric dipole moment, membrane magnetic moment, biomagnetism, 87.14.Cc, 87.16.D-, 83.85.Hf, 87.50.C-, 87.50.uj, 87.16.dt

## Abstract

It was recently reported that static magnetic fields increase lipid order in the hydrophobic membrane core of dehydrated native plant plasma membranes [Poinapen, Soft Matter 9:6804-6813, 2013]. As plasma membranes are multicomponent, highly complex structures, in order to elucidate the origin of this effect, we prepared model membranes consisting of a lipid species with low and high melting temperature. By controlling the temperature, bilayers coexisting of small gel and fluid domains were prepared as a basic model for the plasma membrane core. We studied molecular order in mixed lipid membranes made of dimyristoyl-sn-glycero-3-phosphocholine (DMPC) and dipalmitoyl-sn-glycero-3-phosphocholine (DPPC) using neutron diffraction in the presence of strong static magnetic fields up to 3.5 T. The contribution of the hydrophobic membrane core was highlighted through deuterium labeling the lipid acyl chains. There was no observable effect on lipid organization in fluid or gel domains at high hydration of the membranes. However, lipid order was found to be enhanced at a reduced relative humidity of 43%: a magnetic field of 3.5 T led to an increase of the gel signal in the diffraction patterns of 5%. While all biological materials have weak diamagnetic properties, the corresponding energy is too small to compete against thermal disorder or viscous effects in the case of lipid molecules. We tentatively propose that the interaction between the fatty acid chains’ electric moment and the external magnetic field is driving the lipid tails in the hydrophobic membrane core into a better ordered state.

## 1. Introduction

Magnetic fields are known to interact with biological systems in various ways. Animals, such as pigeons and certain ants, use magnetic fields for orientation [1,2,3,4]. Magnetotactic bacteria move along the direction of an external applied $\vec{B}$ field [5,6]. Biological systems typically show a weak diamagnetism [7], which was used to levitate live animals, such as a grasshopper, mouse and frog [8]. Weak static magnetic fields of 0.2 T were found to induce alterations on human skin fibroblasts [9] and phospholipid bicelles are known to align in an external magnetic field [10,11,12].

While magnetic fields in nature are typically weak (the Earth’s magnetic field is in the order of ∼50 μT), it is possible that strong artificial fields may have a physiological effect. Magnetic fields of 3 T are routinely generated for magnetic resonance imaging (MRI); the latest generation MRI machines use fields of 4.7 T and even 7 T to gain unprecedented spatial resolution.

In plants, more specifically in their seeds, enhanced germination was reported after exposure to magnetic fields [13,14,15]. The germination process starts by water uptake and is accompanied by electrolyte leakage due to seed membrane impairment [16]. It was recently reported that static magnetic fields interact with native plant plasma membranes [17] by increasing lipid order inside of the hydrophobic membrane core.

**Figure 1:**
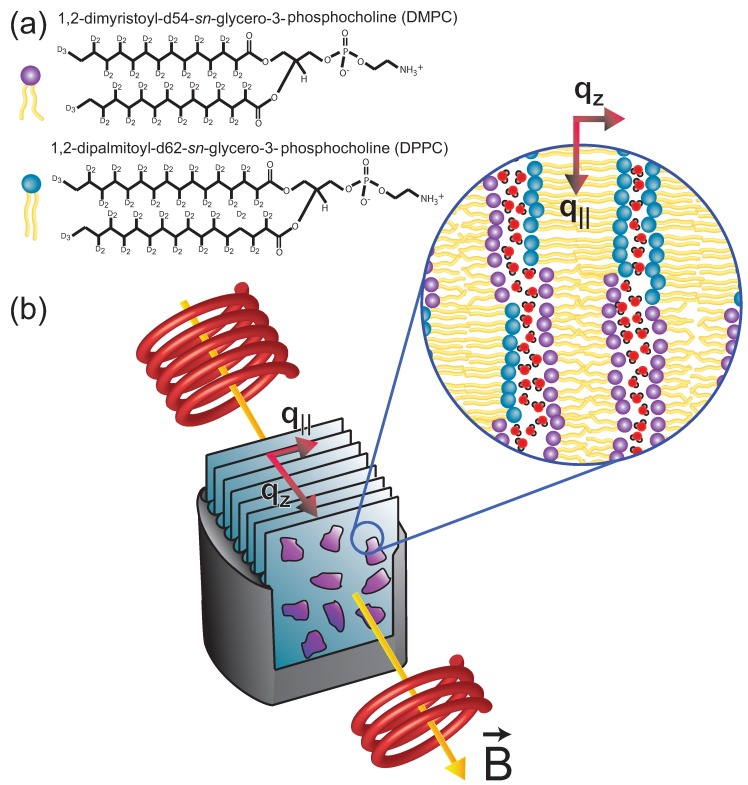
(**a**) Schematic representations of the DMPC and DPPC molecules used to prepare the synthetic membranes for this study. (**b**) Sketch of the scattering geometry: Highly oriented, multi- lamellar membranes were applied on silicon wafers. Eighteen such wafers were mounted inside of a 3.5 T horizontal cryomagnet for the neutron diffraction experiments. Out-of-plane and in-plane structures of the membrane were studied separately but simultaneously. The magnetic field vector, $\vec{B}$, was parallel to the out-of-plane and in-plane scattering vectors, $q_{z}$ or $q_{\left|\right|}$.

The plasma membrane was identified as a potential target for magnetic interactions in this study. However, since the plasma membrane is a complex structure with many different components, we prepared a simplified system to investigate the potential origin of the observed effect. In order to mimic a membrane core of lipids in their gel and fluid state, we mixed dimyristoyl-sn-glycero-3-phosphocholine (DMPC), a 14 carbon saturated phospholipid with a transition temperature of about room temperature, and dipalmitoyl-sn-glycero-3-phosphocholine (DPPC), a 16 carbon saturated phospholipid with a transition temperature of ∼40 ${}^{\circ }$C, at a concentration of 1:1. The molecules are sketched in Figure 1a.

Diffraction patterns were collected at a temperature of 30 ${}^{\circ }$C, between the main transition temperatures of DMPC and DPPC. The molecular structure was studied using neutron diffraction, as sketched in Figure 1b. The bilayers were placed in a cryomagnet to expose them to static magnetic fields of up to 3.5 T *in-situ* during the experiments. By studying the intensity of the gel and fluid signals as a function of hydration and applied magnetic field, we present experimental evidence that static magnetic fields can lead to an increase of the gel signal in dehydrated lipid membranes. By calculating the corresponding energies, we show that this effect is caused not by the membranes’ diamagnetic moment but by the electric dipole moment of the acyl chains.

## 2. Results

Highly oriented, solid supported bilayers made of DMPC and DPPC in a 1:1 ratio were prepared on 1 cm × 1 cm silicon wafers and mounted in an aluminum sample can, which was fabricated to fit into the cryomagnet. The wafers were oriented vertically on a neutron triple-axis spectrometer such that the in-plane structure and the out-of-plane structure could be studied simultaneously simply by rotating the sample by 90${}^{\circ }$ around the vertical *z*-axis, as detailed in the Materials and Methods Section. The cryomagnet produced a horizontal field of up to 3.5 T, which could be changed *in-situ*. The magnetic field vector, $\vec{B}$, was oriented parallel to the scattering vectors, $q_{\left|\right|}$ or $q_{z}$, during the scans, respectively.

The use of neutron beams for this study has the advantage that different parts of the membranes can be deuterium labeled. To emphasize the coherent signal of the hydrophobic membrane core, chain deuterated lipids (DMPC-d54 and DPPC-d62) were used. The neutron triple-axis spectrometer was used in elastic mode such that the monochromator and analyzer were reflecting the same wavelength neutrons.

The experiment was conducted in the following way: we first studied the molecular structure of the DPPC/DMPC membranes for relative humidities between 97% and 10% to explore the effect of magnetic field on gel and fluid domains. The magnetic field dependence was studied in more detail at a relative humidity (RH) of 43%, where $\vec{B}$ was found to increase molecular order in the hydrophobic membrane core.

### 2.1. Membrane Out-of-Plane Structure

**Figure 2:**
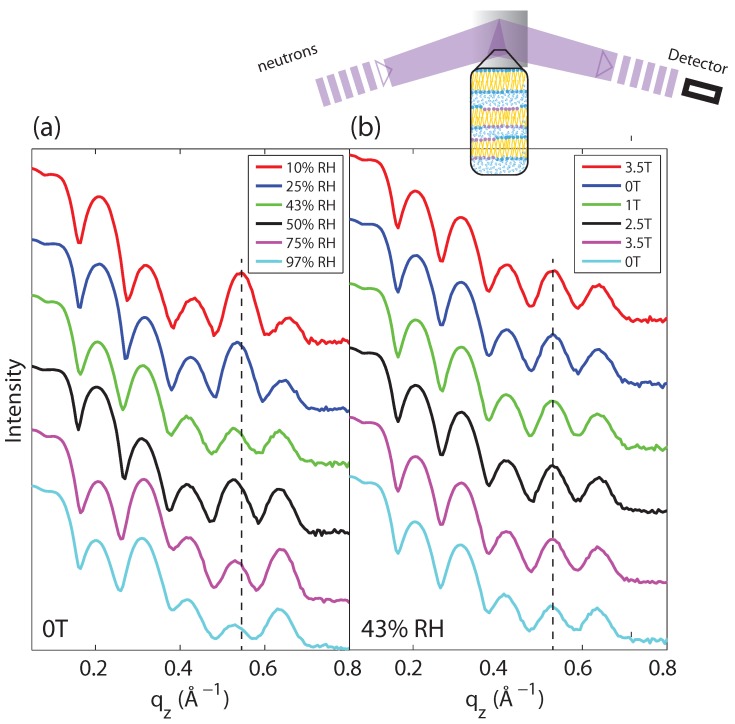
(**a**) Out-of-plane scans for DMPC/DPPC (1:1) membranes measured at different levels of hydration. The well-developed and equally spaced Bragg reflections indicate that the membranes form well-organized lamellar structures. (**b**) Out-of-plane scans at 43% relative humidity at magnetic field strength of 0 T→3.5 T→2.5 T→1 T→0 T→3.5 T. All curves agree within the resolution of our experiment, indicating that the magnetic field does not change the lamellar structure of the membranes.

The quality of the membranes, *i.e*., the lamellar structure of the stacked bilayers, was studied in out-of-plane (reflectivity) scans, and is shown in Figure 2a. Up to five equally spaced and well-developed Bragg peaks were observed for all humidities, which is indicative of a well-organized lamellar structure. The absence of peak splitting indicates that the DMPC/DPPC mixture did not phase separate into DMPC and DPPC bilayers, which would result in two different $d_{z}$ spacings.

Lamellar spacings were determined for all samples and are listed in Table 1. The $d_{z}$ spacings were found to continuously decrease with decreasing hydration, in agreement with previous studies [18,19] in purple membrane and pure DMPC, respectively. We note that $d_{z}$ is the sum of the head-head thickness of the bilayers plus the thickness of the hydration water layer in between the stacked membranes.

Figure 2b shows out-of-plane scans measured at 43% relative humidity for magnetic field strengths of 0, 1, 2.5 and 3.5 T. The fact that the curves at different magnetic fields coincide indicates that magnetic fields do not have an effect on the lamellar organization of the membrane stack (within the resolution of this experiment). We note that the magnetic field vector, $\vec{B}$, was parallel to $q_{z}$ (perpendicular to the bilayers) in these measurements.

**Table 1:** List of all samples prepared for this study. All scans were measured at a temperature of *T* = 30 ${}^{\circ }$C (303 K). Different relative humidities were achieved through saturated salt solutions. Lamellar $d_{z}$ spacings and lipid area of the gel state patches were determined from out-of-plane and in-plane diffraction, respectively.

Lipid Composition	Relative Humidity		Magnetic Field Strength (T)				$d_{z}$ Spacing	Gel Phase Lipid Area (Å${}^{2}$)	
	%	Salt	0	1	2.5	3.5	(Å)	0 T Field	3.5 T Field
DMPC/DPPC (1:1)	97	K${}_{2}$SO${}_{4}$	x	–	–	x	60.7±0.1	43.04	42.98
	75	NaCl	x	–	–	x	58.6±0.7	43.24	43.03
	50	Mg(NO${}_{3}$)${}_{2}$	x	–	–	x	58.5±0.1	42.70	42.70
	43	K${}_{2}$CO${}_{3}$	x	x	x	x	59.0±0.4	42.51	42.51
	25	CH${}_{3}$COOK	x	–	–	x	57.8±0.1	42.23	42.27
	10	LiCl	x			x	56.6±0.1	42.30	42.21

Loss of lamellar order would be observed as a decrease in the intensity of the lamellar peaks, in particular the higher order reflections. A change of the lamellar $d_{z}$ spacing would result in a shift of the Bragg peak positions. While magnetic fields have no observable effect on the out-of-plane structure, changes in the molecular organization in the membrane plane were observed, as will be discussed in the next section.

### 2.2. Membrane In-Plane Structure

**Figure 3:**
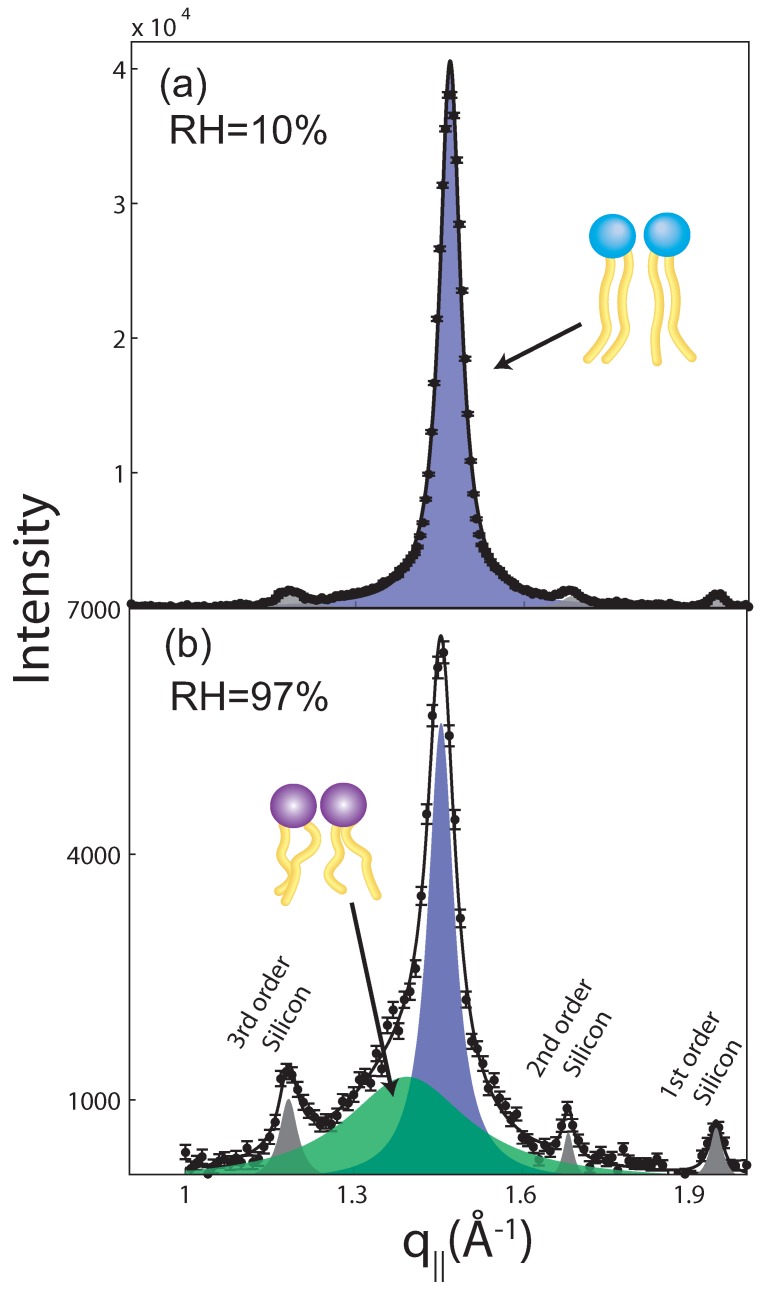
Typical in-plane diffraction at (**a**) 10% relative humidity and (**b**) 97% relative humidity. While the narrow signal at 10% RH indicates that all lipid species take an all-trans (gel) conformation, coexisting gel and fluid signals are observed at high humidity of 97%. Additional signals can be assigned to background scattering from silicon wafers.

Typical in-plane diffraction patterns are shown in Figure 3 for relative humidites of 97% and 10%. The diffraction along the in-plane axis, $q_{\left|\right|}$, shows a number of intensities. Signals at $q_{\left|\right|}$ of ∼1.2 Å${}^{-1}$, 1.65 Å${}^{-1}$ and 1.92 Å${}^{-1}$ can be indexed by higher order background contributions, such as the 3rd order silicon [220], the 2nd order silicon [220] and the 3rd order silicon [111] peak. Lipid signals are observed at $q_{\left|\right|}$ values of 1.39 and 1.46 Å${}^{-1}$. These correlation peaks are well-known as fluid and gel acyl chain correlation peaks [20,21,22], and can be fitted with Lorentzian peak profiles. They correspond to the packing of the lipid acyl chains in the hydrophobic membrane core.

At a low hydration of 10% RH, all lipid species, DMPC and DPPC, are found in their densely packed gel state, as only a gel signal is observed in the data in Figure 3a. At a high hydration of 97%, a broad fluid peak coexisting with a narrow gel peak is observed. The volume fraction of the respective phase is determined from the integrated intensities of the corresponding signals to 1:1. At a temperature of 303 K, 50% of the lipids are above their main phase transition temperature and in their fluid phase (DMPC) while 50% of the lipids are below their main transition and in their gel phase (DPPC), in agreement with the observations.

The lipid tails form a densely packed structure with hexagonal symmetry (planar group p6) in the hydrophobic membrane core, as reported from, e.g., neutron diffraction [23]. The distance between two acyl tails is determined to be $a_{T}=4\pi /\left(\sqrt{3}q_{T}\right)$, which results in a tail spacing of 5.2 Å for the fluid DMPC component and a smaller spacing of 4.9 Å for the better ordered DPPC gel phase. In the absence of strong fluctuations (in the gel state), the area per lipid molecule can be determined from the position of the in-plane correlation peak to $A_{L}=16\pi^{2}/\left(\sqrt{3}q_{T}^{2}\right)$ [24,25,26], or $A_{L}=\sqrt{3}a_{T}^{2}$ when using the lipid spacing. Values for the gel phase areas are listed in Table 1. The almost identical values for areas with and without a magnetic field indicate that the magnetic field does not have an observable impact on the area per lipid.

Based on the out-of-plane and in-plane data, we picture the structure of the bilayers as follows: the solid supported DMPC/DPPC bilayers form well-organized lamellar membrane stacks. The individual bilayers in the stack consist of coexisting fluid and gel patches enriched in DMPC and DPPC lipids. If the two lipid species were mixed uniformly, only one average acyl chain distance should be observed, in contradiction to the in-plane scan in Figure 3b. As too small patches of only a few nanometers in size, would be difficult to observe with scattering techniques due to coherent averaging [23,27,28,29,30], the observed patches can therefore be estimated to be most likely in the order of a few micrometers.

**Figure 4:**
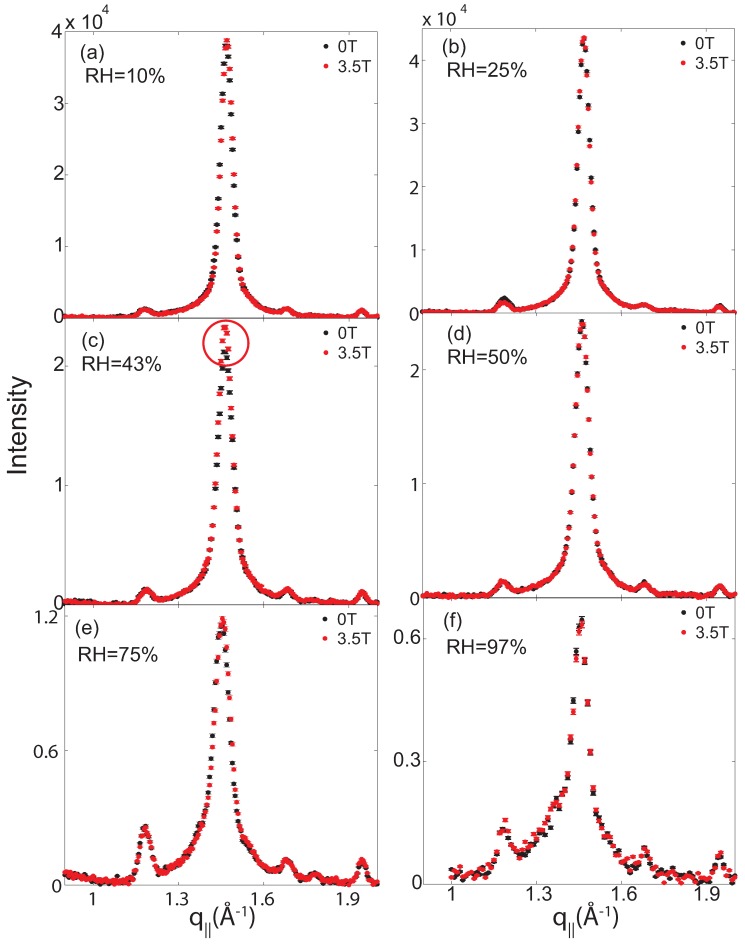
In-plane diffraction patterns for all membranes prepared for this study. Curves measured at 0 T and at 3.5 T are overlaid for an easy comparison. A difference with and without an applied external magnetic field was observed for 43% relative humidity, only. All other curves agree within the resolution of this experiment.

Figure 4 shows in-plane scans measured for all hydration levels. Scans were taken without an external magnetic field, and at a field strength of *B* = 3.5 T. The curves with and without magnetic field all coincide within the resolution of this experiment, except at a relative humidity of 43%. At this humidity, the intensity of the gel peak was found to increase when a magnetic field was present. All scans in Figure 4 were fit using Lorentzian peak profiles for the gel and fluid lipid signals to determine position, width, amplitude and integrated intensity.

**Figure 5:**
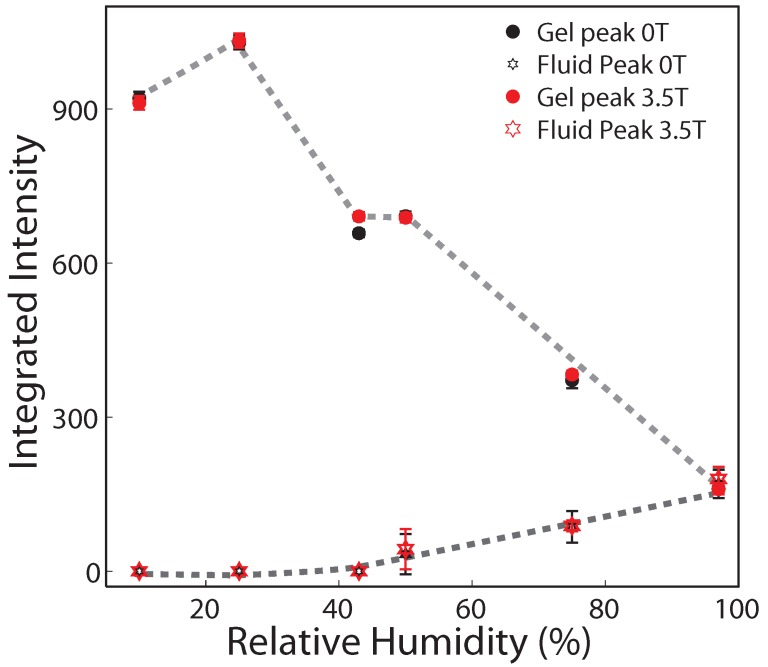
Integrated peak intensities of the gel and fluid correlation peaks from Figure 4 as a function of relative humidity. While gel and fluid peaks have equal intensity at 97% relative humidity, the fluid component reduces with decreased relative humidity and the gel component increases. Intensities for both peaks measured with and without an external magnetic field agree within the resolution of this experiment except at 43% relative humidity. An increase in the gel component with applied field was observed at this relative humidity.

The corresponding integrated intensities for the two signals are plotted in Figure 5. Gel and fluid signals have equal intensity at 97% relative humidity, in agreement with the 1:1 ratio of DMPC and DPPC lipids. When reducing the relative humidity, the fluid signal decreases and the gel signal increases. Except for the relative humidity of 43%, data points with and without magnetic field coincide. We note that the difference in peak intensity at 43% relative humidity was reproducible and well outside the experimental error bars.

**Figure 6:**
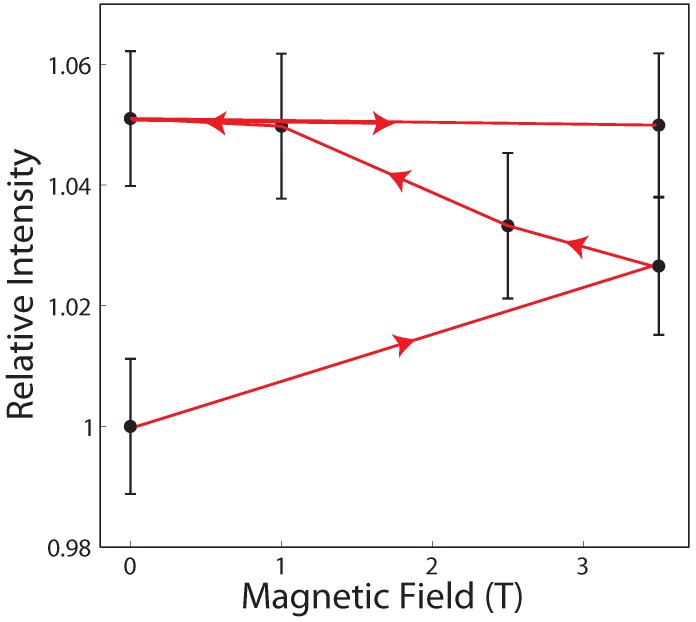
Integrated intensity of the gel signal as a function of magnetic field strength. The magnetic field was changed using the following protocol: 0 T→3.5 T→2.5 T→1 T→0 T→3.5 T. The intensity of the gel signal keeps increasing with time of exposure, rather than being related to absolute field values.

Figure 6 shows the integrated intensity of the gel correlation peak at a relative humidity of 43% as a function of the external magnetic field. Peak areas were normalized to the peak area in zero magnetic field for an easy comparison (the absolute values for the integrated peak intensities are listed in Table 2). The peak was first measured without an external field, *i.e.*, at 0 T, before the field was ramped up to 3.5 T. The field strength *B* was then decreased to 2.5 T, 1 T and switched off again. In the final step of the protocol, *B* was increased back to 3.5 T. The corresponding diffraction patterns were measured for ∼4 h each.

**Table 2:** Integrated intensities from fitting Lorentzian peak profiles to the gel and fluid acyl chain correlation peaks in Figure 4 for all samples. The order of the corresponding field exposure protocol is also given (ord).

	97%			75%			50%			43%			25%			10%		
Field (T)	ord	gel	fluid	ord	gel	fluid	ord	gel	fluid	ord	gel	fluid	ord	gel	fluid	ord	gel	fluid
0	1	161 ± 11	170 ± 27	1	372 ± 15	86 ± 30	1	691 ± 9	33 ± 39	1	648 ± 7	0	1	1029 ± 14	0	1	921 ± 12	0
				3	384 ± 8	89 ± 10				5	691 ± 7	0						
1										4	691 ± 8	0						
2.5										3	680 ± 8	0						
3.5	2	160 ± 10	179 ± 24	2	383 ± 8	88 ± 11	2	688 ± 9	43 ± 39	2	675 ± 8	0	2	1033 ± 14	0	2	911 ± 13	0
										6	691 ± 8	0						

The area of the peak assigned to the gel component increased in total by 5%, well outside of the (conservative) experimental error bars. The increase of the gel correlation peaks indicates an increased order in lipid gel domains in the presence of static magnetic fields at reduced hydration. We note that the intensity did not decrease when the magnetic field was decreased from 3.5 T and eventually switched off. Time of exposure to magnetic fields seems to be more important than the absolute values of the external field. The effects were erased when the sample was placed at higher humidities of greater than 75% RH, or above a temperature of 50 ${}^{\circ }$C.

## 3. Discussion

The aim of this study was to elucidate a potential origin for the effect of static magnetic fields on plant plasma membranes observed by Poinapen *et al.* [17]. In this experiment, the authors observed an increase in the lipid gel signals in dehydrated plasma membranes and identified the membrane core as potential target for the interaction with magnetic fields. We prepared and studied a simplified system by mixing two lipid species, DMPC and DPPC, to create membranes consisting of coexisting small gel and fluid patches. By studying the intensity of the corresponding gel and fluid acyl chain correlation peaks, the effect of magnetic field on the two phases could be investigated.

Highly oriented mixed lipid bilayers made of DMPC and DPPC were exposed to strong static magnetic fields up to 3.5 T and their nanoscale molecular structures were investigated. The membranes were kept at a temperature of *T* = 30 ${}^{\circ }$C and under different relative humidities. Humidity could not be changed *in-situ* continuously, however; experiments were performed at relative humidity values available through different saturated salt solutions. As the temperature was kept between the main transition temperatures of DMPC and DPPC, 50% of the hydrated bilayer consisted of fluid patches and 50% of lipid patches in their gel state at high humidities.

### 3.1. Magnetic Fields Have an Effect on Dehydrated Lipid Membranes

As a first finding, the magnetic fields did not have an effect on the lamellar organization of the membrane stacks. Reflectivity curves at all humidities with and without magnetic field were found to coincide.

No effect of even the strongest magnetic field of 3.5 T could be observed on fluid (made of DMPC) and gel domains (consisting of DPPC) at a high hydration of the bilayers, at 97% relative humidity. Within the resolution of our experiments, static magnetic fields do not seem to affect membrane structure in the physiologically relevant fluid phase of the bilayers. Thermal fluctuations are most likely dominant and we refer to this state as the “fluctuation regime”.

As Poinapen *et al.* reported an effect of magnetic fields in dehydrated plasma membranes, we then started to reduce the hydration of the bilayers. Differences in the in-plane diffraction patterns were observed at a reduced relative humidity of 43%. The correlation peak assigned to the gel lipid component of the membrane were found to increase in intensity in the presence of a magnetic field, in agreement with the observations by Poinapen *et al.*

The magnetic fields were found to have no effect on the membranes at very low levels of hydration of 10% relative humidity, most likely because molecular degrees of freedom are significantly hindered and slowed down; we refer to this state as the “viscous regime”. It is well known that lipid bilayers form more densely packed structures at low relative humidities [31] where fluctuations and molecular motions are strongly suppressed.

The ratio between lipids in their fluid and gel state was determined from the ratio of integrated intensities of the fluid peak to the gel-phase peaks in Figure 5. Following this data, the fluid fraction continuously decreased with decreasing humidity; no fluid lipids were observed at 43% relative humidity. At this humidity, the membranes consisted of DMPC and DPPC patches in their gel state. A static magnetic field directed parallel to the plane of the bilayers was found to increase the integrated intensity of the gel signal by ∼5% at this humidity.

While the integrated intensity of the gel and fluid correlation peaks is proportional to the volume fractions of the respective phases, the increase in gel signal in the presence of magnetic field cannot be attributed to converting fluid into gel lipids, as no fluid phase was observed without a magnetic field. The increase in intensity must be the result of a conformational change of the lipid acyl chains in the hydrophobic membrane core. While the area per lipid molecule is one of the order parameters that affects lipid phases in bilayers, gel lipid areas were found to be unchanged by magnetic fields, as listed in Table 1. This is indicative of a direct interaction between the external magnetic field and the lipid tails in the hydrophobic membrane core.

Our results are in agreement with the results reported by Poinapen *et al.* [17] that static magnetic fields increase ordering in plasma membranes and lead to an increase in the gel signals of the structural lipids. We note that the changes in the corresponding signals observed by Poinapen *et al.* were significantly larger (600%) than the relatively small changes observed in our study. We attribute this to a different experimental protocol: the plasma membranes were exposed to small fields for a significantly longer period of time as compared with our study.

The magnetic fields in our experiment could be changed in-situ to investigate the reversibility of the magnetic field effect, *i.e*., if the structure returns to its initial state when the magnetic field is switched off. This is important to understand whether the effect of the field is to facilitate the kinetics of a transition into a better ordered lipid phase, which is slow the absence of the field, or if the field is moving the system to a new equilibrium state, *i.e*., a thermodynamic state.

The measurements in Figure 6 unambiguously show that the bilayers did not return to their initial state during the time scale of our experiment of a few hours when the magnetic field was switched off. This indicates that the system is trapped in a state with a long relaxation time. Time of exposure seems more important than the actual field strength and the data show a saturation effect: while there is a 3.5% increase when the magnetic field is initially ramped up from 0 to 3.5 T, no effect was observed when the field was ramped up from 0 to 3.5 T again at the end of the procedure. The intensity of the gel signal saturated at a total, maximum increase of ∼5%.

### 3.2. A Potential Mechanism for Lipid Tails to Interact with Magnetic Fields

The reason that the magnetic interaction is observed at 43% RH could be (1) the result that thermal fluctuations and effects due to dehydration cancel out at this relative humidity, such that the small effect of the magnetic field becomes visible, or (2) a resonance effect where the energy related to the magnetic field is of the same size as an energy barrier for molecular motions in the bilayers.

There are several ways a lipid bilayer can interact with a magnetic field. A small diamagnetism is common in most biological materials [7]. An anisotropy of the magnetic susceptibility is used for instance to align phospholipid bicelles in an external magnetic field [10,11,12]. In a magnetic field, the molecule develops an orientation-dependent magnetic moment that interacts with the field producing a torque, which tends to align the molecule consistent with the minimum free energy orientation. For a single molecule, however, this torque is very small and probably cannot compete with thermal disorder [32]. Lipid bilayers, however, may show cooperativity, allowing the net torque to override thermal motion and bring about the alignment of all individual molecules or micellar units. Also, the system must be sufficiently mobile to order on time scales acceptable to laboratory operations. At high viscosities, the rates of reordering required to achieve the minimum energy configuration are prohibitively small, whereas at very low viscosities there may be insufficient cooperativity [32].

The strength of the magnetic dipole moment of a lipid tail can be estimated. The diamagnetic susceptibility of the membrane core was determined to be $\chi =10\times 10^{-6}$ [7]. The dipole moment per unit volume is defined by:

(1) $$\frac{\mu_{m}}{V}=\chi B$$

The volume of a lipid tail is given by half of the lipid area (as listed in Table 1) and the head group-head group distance (typical values for $d_{HH}$ are 40 Å), $V_{T}=A_{T}\;d_{HH}$, to ∼430 Å${}^{-3}$, such that the dipole moment at a magnetic field strength of 3.5 T is determined to be $\mu =1.5\times 10^{-32}$ Am${}^{2}$. The potential energy of such a dipole moment in the magnetic field of 3.5 T is $E_{m}=\mu_{m}B=5\times 10^{-32}$ J. When compared to thermal energy at *T* = 30 ${}^{\circ }$C of $E_{thermal}=k_{B}\;T=303K\times 1.38\times 10^{-23}J/K=4\times 10^{-21}$ J, the magnetic contribution of a single lipid tail to the total energy turns out to be negligible. Even strong artificial magnetic fields are not expected to order lipid acyl chains. The magnetic dipole moment can, however, work to align membranes or bicelles in a magnetic field, when the individual lipids’ moments add up.

Depending on their structural properties, biological molecules and functional groups may also carry an electric dipole moment, $\mu_{el}$, and electric potentials and dipole moments of lipid mono- and bilayers are well known [33,34,35,36]. In particular, phospholipid fatty acid chains contribute to the membrane’s dipole moment [37].

A molecular electric dipole is associated with charge separation along a molecule. When these molecules move in local potentials, the electric dipoles can lead to small currents, which interact with magnetic fields through the Lorentz force, ${\vec{F}}_{L}=q\;\vec{v}\times \vec{B}$.

The torque on a fluctuating electric dipole is defined by:

(2) $${\vec{\tau }}_{el}={\vec{\mu }}_{el}\times \left(\vec{v}\times \vec{B}\right)$$

The minimum energy orientation of an electric dipole in an external magnetic field is thus perpendicular to the field direction and perpendicular to the direction of the fluctuation ($\tau_{el}$ is zero in this case). The ballistic velocity of lipid molecules (at *T* = 310 K) has recently been reported to be *v* = 87 m/s [38], which can serve as an estimate of molecular velocities. The contribution of CH${}_{2}$ bonds in the lipid tails to the membrane’s electric dipole potential Δϕ was determined to be 20 mV [37]. The corresponding electric dipole moment can be determined from the relation $\Delta \phi =12\pi \mu_{el}/A_{T}$ to $\mu_{el}=1.1\times 10^{-22}$ Cm.

With these numbers, $\tau_{el}$ is calculated to be $\tau_{el}=3.3\times 10^{-20}$ Nm. The energy related to tilting the electric dipole moment by an angle *α* of, say, *α* = 10${}^{\circ }$ is then:

(3) $$W_{el}=\alpha \;\tau_{el}=10\;\times \;3.3\times 10^{-20}\;Nm=3.3\times 10^{-19}\;J$$

**Figure 7:**
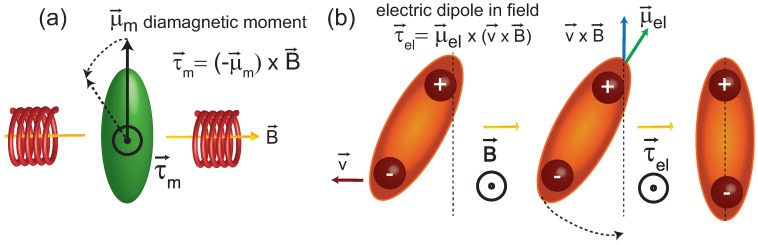
Interaction between a magnetic moment (**a**) and an electric dipole moment (**b**) and an external magnetic field. The magnetic moment experiences a torque, $\tau_{m}$, towards the direction of the magnetic field. The torque on a fluctuating electric dipole is acting to align it with the bilayer normal. While the magnetic energy $E_{m}=\mu_{m}B=5\times 10^{-32}$ J is small as compared to thermal energies ($E_{thermal}=k_{B}\;T=4\times 10^{-21}$ J), the energy related to a 10${}^{\circ }$ tilt of the electric dipole is calculated to $W_{el}=\alpha \;\tau_{el}=3.3\times 10^{-20}\;J$, comparable to $E_{thermal}$. See text for details

This energy is in the order of thermal energies and may, therefore, be relevant for the observed effect. The different mechanisms are pictured in Figure 7.

The direction of this torque is parallel to the direction of the applied magnetic field and perpendicular to $\vec{v}$. The resulting motion is a fluctuating torque of the lipid tails acting in the direction of the membrane normal. While a magnetic moment would lead to molecular tilts out of the membrane normal, an electric dipole moment aligns the tails along the bilayer normal, which may serve to order lipid tails and further suppress lipid tail fluctuations. The corresponding signal should show an increase in diffraction experiments, as it was observed in the neutron diffraction data.

We note that it is assumed that all chain segments contribute to the electric dipole moment in the above calculation, implying straight, well-ordered lipid tails, typical for lipid molecules in their gel phase. The introduction of gauche defects in the fluid phase may lead to smaller, more randomly oriented values of the torques on different chain segments such that the overall effect may cancel out. At very low hydrations, the bilayers form densely packed structures and the molecular motions are strongly suppressed. The electric torque will be reduced the same way fluctuations decrease. In addition, the energy to orient molecules or segments will be drastically increased such that much stronger magnetic fields would be needed to overcome the corresponding energy barriers.

A magnetic field is, therefore, not likely to have an observable effect on the fluctuation regime, at high humidities, or on the viscous regime, at very low hydrations. At intermediate levels of hydration, however, the fluid phase is suppressed and better ordered gel phases form with well-aligned lipid acyl chains, which may be more susceptible to the ordering effect of an external magnetic field. In the case of DMPC/DPPC membranes, this level of hydration is found at ∼43% relative humidity using saturated K${}_{2}$CO${}_{3}$ salt. As humidity could not be controlled continuously in our experiment, at this point the optimum humidity cannot be determined with higher precision.

While we present experimental findings and a first observation in this study, several questions remain open. Future studies will measure the response of the bilayers at 43% RH relative to magnetic fields in more detail, as functions of field strength, protocol of exposure, potential history effects and directional dependence. By using a setup that allows to control humidity continuously, the dependence of the effect could be determined more precisely and humidities around the maximum could be explored. It will also be interesting to prepare bilayers made of saturated and unsaturated lipid species, which should stay fluid even at lower levels of hydration, to investigate the effect of magnetic fields on fluid phase lipids in more detail.

## 4. Conclusions

We studied the effect of strong external static magnetic fields of up to 3.5 T on lipid organization in phospholipid membranes made of DMPC and DPPC. By setting the temperature between the main transition temperatures of the two lipid species, patchy bilayers consisting of small gel and fluid lipid patches were created to study the effect of the magnetic field on the respective phases. We measured the changes in the molecular organization as a function of relative humidity and magnetic field strength using neutron diffraction. The experiments were mainly sensitive to the contribution of the hydrophobic membrane core by using selective deuteration and chain deuterated lipid molecules.

The magnetic field did not have an observable effect on the lamellar structure of the membrane stacks. No effect of magnetic fields on gel or fluid phases was observed at a either a high hydration or very low hydration of the membranes. However, at a relative humidity of 43%, the gel signal was found to increase by 5% in the presence of a 3.5 T external magnetic field directed in the plane of the bilayers. The effect was found to depend on the time of exposure rather than the absolute strength of the magnetic field.

The diamagnetic interaction energy between lipid tails and magnetic field was found to be too small to compete against thermal disorder or viscous effects in the case of a single lipid molecule. However, the interaction between the fatty acid chains’ electric dipole moment and the external magnetic field can likely drive the hydrophobic membrane core into a better ordered state. This state is metastable and decays slowly with time after the magnetic field is switched off.

## 5. Materials and Methods

### 5.1. Neutron Diffraction

**Figure 8:**
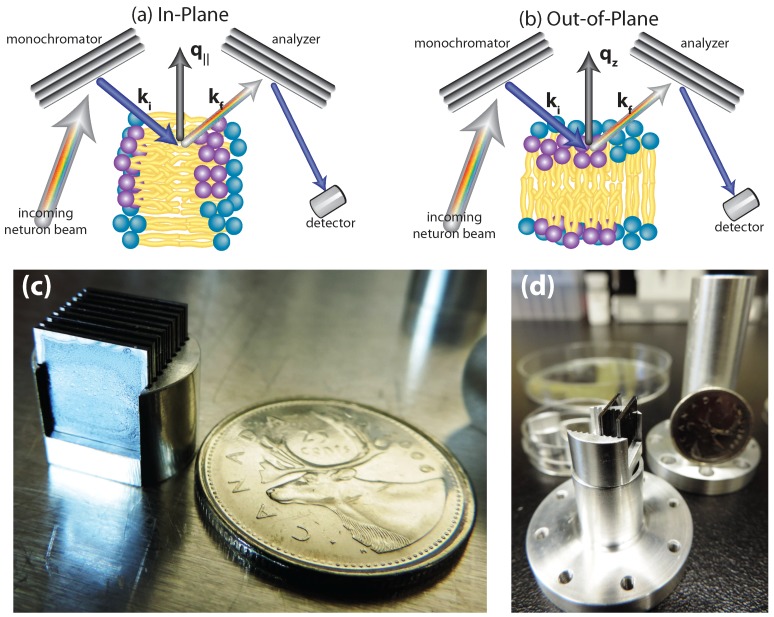
(**a**) and (**b**): Sketch of the scattering geometry to measure in-plane and out-of-plane structure using a triple-axis spectrometer. (**c**) Photo of the solid supported membranes on silicon wafers mounted in a fabricated holder. (**d**) Photo of the aluminum sample can, which was inserted into the cryomagnet. Saturated salt solutions were added to the container underneath the samples to achieve different levels of hydration.

Experiments were conducted using the cold triple-axis spectrometer IN12 at the high flux reactor at the Institut Laue-Langevin (ILL) in Grenoble, France. The three axes of the spectrometers refer to the axis of rotation of the monochromator, the sample and the analyzer. The incident and final neutron energies are defined by the Bragg reflections from pyrolytic graphite (PG) crystals. No collimation was used but monochromator and analyzer were focused to maximize the number of incident neutrons on the membranes. A schematic of the instrument configuration is shown in Figure 8a,b. In-plane and out-of-plane structure can be measured simultaneously on a TAS by simply rotating the sample by 90${}^{\circ }$.

The sample can was installed into a 3.8 T horizontal field cryomagnet. A neutron wavelength of 0.418 nm was used. This wavelength is beyond the aluminium and silicon cut-off such that the corresponding Bragg reflections cannot be excited, at least not the first order, which decreases background contribution and enhances the signal-to-noise ratio. All experiments were conducted at 30 ${}^{\circ }$C, (T = 303 K). On a triple-axis spectrometer the analyzer cuts out only the elastically scattered neutrons. The quasi-elastic contributions of the protons to the background are omitted, further reducing the background and improving the signal-to-noise ratio drastically. The combination of a low background, good *Q*-resolution, the use of an analyzer, and the option for a powerful horizontal magnet made IN12 highly suitable for diffraction experiments in membranes.

The static structure factor S(Q) was determined in external magnetic fields of 0, 1, 2.5 and 3.5 Tesla. In order to avoid the risk of quenching the magnet, the highest achievable field of 3.8 T was not used. The scattering vector (either $q_{z}$ or $q_{\left|\right|}$) was placed parallel to the magnetic field lines such that $\vec{B}$ was in the plane of the membranes for the in-plane diffraction scans and perpendicular to the bilayers for the out-of-plane scans. We note that it was technically not possible to measure out-of-plane diffraction with an in-plane field because of the design of the cryomagnet used.

### 5.2. Membrane Preparation

Highly oriented multi-lamellar stacks of 1,2-dimyristoyl-sn-glycero-3-phosphocholine (DMPC) and dipalmitoyl-sn-glycero-3-phosphocholine (DPPC) were prepared on 1 cm×1 cm, 300 μm thick, single-sided polished Si wafers. The coherent scattering of the lipid hydrocarbon chains was enhanced by using tail deuterated lipids, *i.e*., DMPC-d54 and DPPC-d62. A 20 mg/mL suspension of DMPC-d54 and DPPC-d62 in 1:1 chloroform and 2,2,2-trifluoroethanol (TFE) was prepared. The Si wafers were cleaned by alternating 30 min sonications in dichloromethane (DCM) at 293 K to remove all organic contamination and leave the substrates in a hydrophilic state. This process was repeated twice. The wafers were then thoroughly rinsed three times using ∼50 mL of ultrapure water and methanol alternatingly. The cleaned wafers were placed on a heated sample preparation surface, which was kept at 40 ${}^{\circ }$C (313 K). This temperature is above the main phase transition temperature of DMPC and DPPC, thus the heated substrates ensured that the lipids were in the fluid phase during deposition and the self-assembly of the lipids. An 80 μL aliquot of the lipid solution was deposited on each Si wafer in a titling incubator, which was set to a speed of 15 rev/min and tilt of 1${}^{\circ }$ to allow the lipid solution spread evenly across the wafer. The temperature was kept at 313 K and the solvent was allowed to slowly evaporate for 10 min. The wafers were kept in vacuum overnight to remove all traces of the solvent and incubated with heavy water, D${}_{2}$O, at 313 K for 24 h. Following this protocol, each wafer contained ∼3000 highly oriented membranes, which was about 10 in total thickness.

Eighteen sample-containing Si wafers were mounted in an aluminium sample holder fabricated to be inserted into the 3.8 T horizontal field cryomagnet available at the ILL. A photo of the sample and the aluminum sample holder is shown in Figure 8c,d. Hydration of the lipid membranes from the vapor phase was achieved by a water reservoir in the bottom of the sample holder. The well in the aluminum sample can was filled with saturated solutions of different salts, as listed in Table 2, and the membranes were hydrated to the respective hydration levels from the vapor phase. Discrete values for the relative humidity could be prepared using this protocol. The lamellar repeat spacings achieved with this setup are listed in Table 1. Between measurements of different humidities, samples were placed in an incubator at 75% RH and 50 ${}^{\circ }$C to re-anneal the structure and erase a potential memory to ensure that the starting point for all the scans remained the same.

The samples were mounted vertically in the neutron beam such that the scattering vector ($\vec{Q}$) could either be placed in the plane of the membrane ($\vec{q_{\left|\right|}}$) or perpendicular to the membrane ($\vec{q_{z}}$). Out-of-plane and in-plane structure could be measured by simply rotating the sample by 90${}^{\circ }$.

The temperature of the main transition in fully hydrated bilayers made of DMPC was reported at *T* = 296.6 K in multi-lamellar DMPC systems [18,19,39]. In multilamellar DMPC-d54 bilayers the transition from the gel into the fluid phase occurs at a slightly lower temperature of $T_{m}$ = 21.5 ${}^{\circ }$C = 294.7 K [22,40]. The main transition temperature, $T_{m}$, of DPPC-d62 was reported to occur at 310.5 K [41], a value slightly lower than its protonated counterpart (*T* = 314.4 K) [41,42].
